# HPV vaccines in Brazil and the world

**DOI:** 10.1186/1753-6561-8-S4-O7

**Published:** 2014-10-01

**Authors:** Luisa Villa

**Affiliations:** 1 School of Medicine, University of São Paulo, São Paulo, Brazil

## 

Human papillomavirus (HPV), the most common newly acquired, sexually transmitted infection worldwide, infects about 75% of sexually active individuals in their lifetimes. The high-risk HPV types, the most common of which are HPV 16 and 18, cause cervical and other anogenital cancers, as well as some head and neck cancers [1]. Different HPV types are related to different lesion grades. HPV16 is found in about half of cervical cancers, the majority of anal cancers and about 30% of oropharyngeal cancers, besides a significant proportion of vulvar, vaginal and penile carcinomas. The low-risk types, the most common of which are HPV 6 and 11, cause genital warts, low-grade cervical dysplasias and recurrent respiratory papillomatosis. Most of our understanding of HPV and carcinogenesis is derived from studies of women and cervical cancer. However, it is clear that HPV causes a significant proportion of tumors in both women and men [2]. Therefore, new prophylactic HPV vaccines have the power to prevent many HPV infections, thus reducing the burden of HPV-associated diseases.

Two vaccines have been developed, a quadrivalent vaccine that protects against HPV 16, 18, 6 and 11 (Merck & Co., USA) and a bivalent vaccine that protects against HPV 16 and 18 (Glaxo Smith-Kline, Belgium). Both vaccines are composed of HPV L1 proteins that have spontaneously self-assembled into virus-like particles (VLPs). However, they have different manufacturers, valencies, adjuvants and are produced in different types of cells. Both are administered by intramuscular injection, in three doses (0, 1 or 2 and 6 months). In a number of clinical trials conducted in thousands of subjects in many countries since 1998, the safety, immunogenicity and efficacy of these HPV VLP vaccines have been demonstrated [3].

Prophylactic efficacy was measured considering HPV infection and disease endpoints, particularly high-grade cervical intraepithelial neoplasia or worse for the bivalent and quadrivalent vaccines, as well as vulvar intraepithelial neoplasia (VIN) or vaginal intraepithelial neoplasia and genital warts, for the quadrivalent vaccine only. For both the bivalent and quadrivalent vaccines, results of different trials allow for the examination of broad trends in efficacy in preventing HPV 6/11/16/18-related disease in several groups of patients categorized according to their HPV status at baseline. Very high efficacy rates were observed in naive women for the vaccine-HPV types which led to the recommendation of the vaccine to young individuals, prior to exposure to HPV which occurs concomitant to sexual activity onset. Cross-protection against non-vaccine types was also noted, but the significance and durability of protection is yet to be defined. To increase the coverage to more than 70% of cervical cancers attributable to HPV 16 and 18, it is possible that the best and most efficient responses will be obtained with multivalent vaccines against a larger number of genital, anal and oropharyngeal HPV infections which are presently under development [4].

VLPs are noninfectious protein subunit vaccines and show safety profiles similar to other protein subunit vaccines such as tetanus or hepatitis B vaccines. Both vaccines were generally well tolerated in clinical trials and maintain a good safety profile after licensure. Since the VLP vaccines were designed primarily to protect by inducing virion neutralizing antibodies, type specific antibody responses to the VLPs have been the primary focus of immunogenicity studies. Both vaccines were shown to be highly immunogenic in the clinical trials, resulting in essentially 100% seroconversion in the different populations studied which included both females and males. Both vaccines induce the expected B cell memory response which is a property of vaccines with durable immune responses. Although the long term persistence of stable antibody levels is an encouraging finding, the antibody levels needed to prevent infection or disease are currently unknown. However, long-term follow-up studies have shown that efficacy is maintained for at least ten years and modeling studies suggest protection may last much longer. Available data do suggest that HPV vaccines may provide a lengthy period of protection, likely to usher a vaccinated individual through the years of highest infection risk and beyond. Additional studies are ongoing to verify these projections.

*HPV vaccines introduction and implementation*. Based on its demonstrated clinical efficacy and favorable safety profile, HPV prophylactic vaccines have been introduced in hundreds of countries around the world, including most countries in Latin America. In Brazil, the quadrivalent HPV vaccine was approved in 2006 and the bivalent vaccine in 2008 [5]. The rapid approval and launch of such vaccines are a clear indication that governments and policy makers are aware of the expected impact on the prevention of one of the most common causes of female mortality worldwide.

In 2007, Australia was one of the first countries to introduce the quadrivalent HPV vaccine in a public immunization programme for girls and young women. Four years after the beginning of the HPV vaccination program, a substantial decrease in vaccine-targeted genotypes is evident. In fact, a dramatic reduction in genital warts was observed in young women as well as young men, an indication of herd immunity [6]. On the other hand, older women and men, as well as men who have sex with men, show very little or no reduction of this benign tumor caused by low-risk HPV types. These results led Australia to include young men in their national immunization program which, in time, translate into reductions in HPV-related lesions in both genders. Moreover, a reduction in high-grade cervical abnormalities is being observed as a consequence of the introduction of HPV vaccination [7]. Similar results are rapidly accumulating in several countries which implemented national HPV vaccination programs including New Zealand, Sweden, Denmark, Belgium, Germany, Canada, and USA. Despite such very positive results, incorporation of HPV vaccination in the public health sector is still to be seen in the developing world, mostly due to vaccine cost. HPV vaccine implementation will also depend on local infrastructure for vaccine delivery to the initial target population during the window of highest vaccine efficacy, i.e., prior to sexual exposure. Furthermore, introducing HPV vaccines in the present cervical cancer control system is hampered by the fact that secondary screening with Pap tests (or HPV DNA testing) will still be required to detect cervical cancers and pre-cancers caused by non-vaccine HPV types [8]. Ongoing cost-benefit studies and negotiations between governments, the private sector, and non-governmental organizations, are enabling several developing countries, where the vaccine is most needed, to implement the necessary programs [9]. The Ministry of Health of Brazil has announced in June 2013 the decision to introduce the quadrivalent HPV vaccine in the national immunization program. From 2014, this vaccine will be offered to girls and adolescents 11-12 years old. The details of the program are largely unknown which includes the possibility of an extended vaccination schedule (0, 6, 60 months; in clinical trials 0, 2 6 months).

The success of the prophylactic vaccines against HPV is clear. However, these vaccines are not and will not be available in the short run, as public immunization program in many countries. Moreover, the coverage rates will vary at large due to economic restrictions and other issues. Vaccine acceptance is largely de-termined by health beliefs, such as the individual's perceived susceptibility to the disease, vaccine characteristics, such as cost and efficacy, and obstacles to obtaining the vaccine. Education of physicians, policy makers, parents and adolescents will be crucial for delivering HPV vaccines to target populations during the window of highest vaccine efficacy, prior to sexual debut. Despite all efforts and the excellent results observed in real life, it may take some time before these vaccines are administered to the general population worldwide. Moreover, women will still be at risk for developing cancers caused by other HPV types not included in the vaccine and hence screening and monitoring strategies will be required. It is important to stress that disease outcomes should be recorded since at present there is no immune correlate and the importance of serum antibody levels is simply not known. Moreover, vaccinated populations should be followed-up for long-term safety, sustained immune responses and vaccine disease efficacy [10].
